# Age- and sex-specific effects in paravertebral surface electromyographic back extensor muscle fatigue in chronic low back pain

**DOI:** 10.1007/s11357-019-00134-7

**Published:** 2019-11-27

**Authors:** Gerold Ebenbichler, Richard Habenicht, Sara Ziegelbecker, Josef Kollmitzer, Patrick Mair, Thomas Kienbacher

**Affiliations:** 1 Department of Physical Medicine, Rehabilitation and Occupational Medicine, Medical University of Vienna, Vienna General Hospital, Währinger Gürtel 18-20, 1090 Vienna, Austria; 2 Karl-Landsteiner-Institute of Outpatient Rehabilitation Research, Vienna, Austria; 3 Department of Psychology, Harvard University, Cambridge, MA USA; 4 Technical School of Engineering, Vienna, Austria

**Keywords:** Muscle fatigue, Low back pain, Surface electromyography, Aging

## Abstract

The impact of aging on the back muscles is not well understood, yet may hold clues to both normal aging and chronic low back pain (cLBP). This study sought to investigate whether the median frequency (MF) surface electromyographic (SEMG) back muscle fatigue method—a proxy for glycolytic muscle metabolism—would be able to detect age- and sex-specific differences in neuromuscular and muscle metabolic functions in individuals with cLBP in a reliable way, and whether it would be as sensitive as when used on healthy individuals. With participants seated on a dynamometer (20° trunk anteflexion), paraspinal SEMG activity was recorded bilaterally from the multifidus (L5), longissimus (L2), and iliolumbalis (L1) muscles during isometric, sustained back extensions loaded at 80% of maximum from 117 younger (58 females) and 112 older (56 female) cLBP individuals. Tests were repeated after 1–2 days and 6 weeks. Median frequency, the SEMG variable indicating neuromuscular fatigue, was analyzed. Maximum back extensor strength was comparable between younger and older participants. Significantly less MF-SEMG back muscle fatigue was observed in older as compared to younger, and in older female as compared to older male cLBP individuals. Relative reliability was excellent, but absolute reliability appeared large for this SEMG-fatigue measure. Findings suggest that cLBP likely does not mask the age-specific diagnostic potential of the MF-SEMG back extensor fatigue method. Thus, this method possesses a great potential to be further developed into a valuable biomarker capable of detecting back muscle function at risk of sarcopenia at very early stages.

**Electronic supplementary material:**

The online version of this article (10.1007/s11357-019-00134-7) contains supplementary material, which is available to authorized users.

## Introduction

It is widely accepted that muscle weakness is a major risk factor for low back pain (LBP). This is particularly true in older individuals who commonly experience both back extensor muscle weakness and chronic LBP (cLBP) (GBD Disease and Injury Incidence Prevalence Collaborators 2018; Hoy et al. 2014). In such individuals, both the burden of back pain and the accompanying changes in movement behavior likely facilitate weakening of back extensor muscles, thereby fueling a vicious circle with bodily inactivity, back muscle wasting, and chronic back pain (Ebenbichler et al. 2001; van Dieen et al. 2018; Vlaeyen et al. 2016). Therefore, it may be hypothesized that back pain facilitates an age-related weakening process within the back extensors, thereby contributing to higher levels of pain-related disablement, early dependency, and the need for institutionalization or even early death (Beauchamp et al. 2016; Suri et al. 2009; Suri et al. 2011). This process may be particularly prevalent in older cLBP individuals. Hence, muscle function-based biomarkers that can identify cLBP patients at risk of early aging and sarcopenia—the latter is defined as a muscle disease rooted in adverse muscle changes that occur across a lifetime (Cruz-Jentoft et al. 2019)—would be of utmost interest in the context of reducing the health burden of sarcopenia through early intervention (Calvani et al. 2017; Ciolac and Rodrigues-da-Silva 2016). However, the sparse research conducted to date does not unequivocally support the idea that muscle function relates to chronic low back pain in aging persons.

Current concepts on the pathomechanisms that lead to sarcopenia are complex, and include the following: a progressive loss of motor neurons; a loss of preferentially type II muscle fibers and muscle fiber atrophy; a loss of muscle fiber elasticity and alterations in muscle structure; a negative muscle protein balance with blunted protein production to anabolic stimuli, accompanied by an increased rate of protein degradation that includes the quantity and quality of contractile proteins and proteins related to the excitation-contraction coupling; a deficient formation and malfunctioning of cell organelles; and impaired activation and differentiation through muscle satellite cells (for review see, Larsson et al. 2019). On the muscle cell function level, a blunted protein production in aged muscles is caused by impaired anabolic signaling of the peroxisome proliferator-activated receptor-ɣ coactivator-1α (PGC-1α)—a key regulator of mitochondrial biogenesis—which may reduce sensitivity to insulin and impair the expression of Akt and mammalian target of rapamycin (mTOR). In parallel, increased muscle protein degradation via the calpain and the autophagy pathways (rather than the ubiquitin proteasome pathway) takes place (Riuzzi et al. 2018). Loss of muscle endurance and strength is related to deficiencies within the respiratory chain due to a decline in both the number and function of mitochondria (Joseph et al. 2016). Overproduction of harmful reactive oxygen molecules caused by decreased activity of anti-oxidant buffers in aged muscles may induce an excessive mutant mitochondrial genome (Bua et al. 2006) and/or a decline in number of mitochondrial DNA copies (Hebert et al. 2015), as well as impair the mitophagy system (Tieland et al. 2018). Impairments in any of these pathways likely result in deficient oxidative phosphorylation and impaired energy production. If homeostasis is not re-established, myonuclear cell death is activated and muscle atrophy ensues (Joseph et al. 2016). Loss of PCG-1 expression in senescent myocytes may further impair the maintenance of the neuromuscular junction (Vainshtein et al. 2015), as well as the connectivity between satellite cells and myocytes following an insufficient stimulation of vascular endothelial growth factor (VEGF) release from myocytes (Arany et al. 2008). Capillary rarefication in senescent muscles is thought to be linked to blunted regenerative capacity and plasticity of satellite cells as well as their anabolic resistance (Nederveen et al. 2016). Hormonal changes with old age, low systemic inflammation, insufficient protein intake, and bodily inactivity, among other factors, may all facilitate wasting, weakness, and early fatigue in senescent muscles of otherwise healthy individuals (Joseph et al. 2016; Larsson et al. 2019; Tieland et al. 2018).

An increasing body of research supports the “cLBP-driven early muscle aging” theory, including back imaging studies that observed a loss in muscle cross-sectional area (a suggested indicator of force production capacity (Lieber and Bodine-Fowler 1993)), and increased intramuscular fat replacement (Dahlqvist et al. 2017; Goubert et al. 2016; Mannion et al. 1998; Sions et al. 2017b) (fat replacement is indicative of aging and pathology (Hebert et al. 2014)). However, such age- and pain-related magnet resonance imaging (MRI) diagnosed muscle morphological alterations were of limited predictive value when related to the degree of disablement (Sions et al. 2017a; Suri et al. 2015). By contrast, observations of divergent alterations in muscle fiber distribution within the back extensors of older individuals and individuals with cLBP widely refute such a theory. Alterations in muscle fiber proportion within a muscle may be regarded as a proxy for changes in the muscles’ capability to produce strength and endurance. Whereas aging back extensor muscles of healthy individuals have been found to have a decreased proportion of the glycolytic type II muscle fiber area (Ng et al. 1998), such alterations revealed higher than normal or similar proportions of glycolytic type II muscle fibers in cLBP when compared to healthy, age-matched controls (Mannion 1999; Mannion et al. 1998). Such divergent findings on histomorphology could even suggest that cLBP might slow rather than facilitate the aging process within back extensor muscles. In addition, as back extensors of healthy females were found to have a lower proportion of glycolytic fibers and an overall higher resistance to back muscle fatigue than those of males, the back muscle aging process in individuals with cLBP may differ between males and females in a striking way (Clark et al. 2003; Mannion et al. 1997; Mannion et al. 1998).

The surface electromyographic (SEMG) fatigue method is widely used to assess back muscle function—i.e., muscle strength and endurance—independent of its maximum effort (De Luca 1997). The median frequency (MF)-SEMG fatigue method—expressed as a time-related shift of the power spectral MF-SEMG toward lower frequencies—recorded from higher loaded muscle contractions has repeatedly been touted as a particularly suitable and objective non-invasive measure of the glycolytic metabolism of back extensor muscles (De Luca 1997). Studies to date have not confirmed which metabolic processes underlie the MF-SEMG fatigue behavior, however, preliminary findings suggest that the fatigue effects are mediated through hydrogen ion accumulation resulting from anaerobic metabolic pathways (e.g., Brody et al. 1991; Kupa et al. 1995). Such studies strongly suggest that MF-SEMG fatigue relates to the proportion of glycolytic type II muscle fibers in both healthy volunteers (Kienbacher et al. 2014a; Merletti et al. 2002; Merletti et al. 1992) and in individuals with a medical condition like back pain (Roy et al. 1997). With advancing age, a lower proportion of fast fatiguing, glycolytic muscle fibers subsequent to loss of preferably highly myelinated axons (e.g., Larsson et al. 2019), would thus relate to less MF-SEMG muscle fatigue. Therefore, lower muscle fatigue rates might allow diagnosis of aging processes within the neuromuscular system at an early stage. It is worth noticing that the MF-SEMG fatigue measure would be widely independent of the maximum strength output as long as the maximum score was obtained from a true maximum contraction (Oddsson and De Luca 2003). This is important, as declines in muscle strength following axonal apoptosis may to a variable extent be compensated through muscle regenerative processes as well as training effects within the remaining, less fatiguing, low threshold motor units (Frontera and Ochala 2015; Frontera et al. 2008; Larsson et al. 2019).

The MF-SEMG fatigue method was found to be a reliable measure of muscle capability, if muscle contractions were performed at strength levels sufficient to allow (1) the complete recruitment of all motor units under the detection field of the electrode and (2) the blood flow to active muscles to be widely occluded (De Luca 1997). When using this spectral SEMG method, clearly less back muscle fatigue was observed in older as compared to younger healthy individuals (Kienbacher et al. 2014a). In addition, when using the spectral SEMG method, more back or gluteal muscle fatigue was observed in persons with cLBP as compared to healthy individuals (da Silva et al. 2015; Kankaanpaa et al. 1998b; Lariviere et al. 2003; Roy et al. 1989; Roy et al. 1990; Sung et al. 2009). The MF-SEMG method was also able to discriminate between cLBP patients who were kinesiophobic fear avoiders, as classified by more muscle fatigue, and endurers, as classified by less muscle fatigue (Biedermann et al. 1991; Lariviere et al. 2010). MF-SEMG has appeared to be sufficiently sensitive to differentiate between female and male back extensor function, with less fatigue resistance in males than females (Clark et al. 2003; Kankaanpaa et al. 1998a). Collectively, if sustained back extensions were performed at submaximal loads that control major sources of bias of the SEMG signal (De Luca 1997; Oddsson and De Luca 2003; Roy and Oddsson 1998), these findings would attest the spectral SEMG fatigue method to be an appropriate, non-invasive proxy for back muscle metabolic function and capability.

Developing novel biomarkers for detecting very early signs of sarcopenia based on surface electromyography will require a more mature understanding of the neuromuscular aging process in male and female individuals. Such knowledge would allow understanding of whether the potential application of this screening test could be expanded from healthy individuals to persons with cLBP. To test this idea, the primary objective of this research was to investigate age- and sex-specific differences in MF-SEMG back muscle fatigue in cLBP patients to shed light on the mechanisms related to neuromuscular capability and muscle glycolytic functioning as males and females age. If this method proves sensitive enough to detect age- and sex-specific effects and is reproducible from day to day, then it may be developed into a screening tool to detect very early signs of aging in back muscles, not only in healthy pain-free individuals (Kienbacher et al. 2014a) but also in those with cLBP. In addition, this measure could potentially be used as a surrogate marker of therapeutic interventions intended to slow down the early and/or accelerated aging of the neuromuscular system in individuals diagnosed to be at an increased risk for sarcopenia at an early stage. We therefore also examined the reliability of the tests from day to day.

## Methods

The study protocol was approved by the Ethics Committee of the City of Vienna. All participants provided written informed consent. The data collection was carried out in accordance with the directives of the Declaration of Helsinki.

### Participants

Over a period of 3 years, study participants were recruited by word of mouth from chronic low back pain patients who were referred to ambulatory rehabilitation (all between 18 and 90 years of age). All persons were presented with the study concept and informed that they would be provided, on a voluntary basis, with 6 months of cost-free training after the testing at the Karl-Landsteiner Institute of Outpatient Rehabilitation Research where the data collection took place.

A total of 294 patients with cLBP (159 females) between 18 and 90 years of age decided to volunteer and consequently completed a short screening questionnaire that assessed the location, duration, and intensity of pain as well as some functional limitations and co-morbidities. Thereafter, eligible persons were scheduled for an examination performed by a Physical and Rehabilitation Medicine specialist. All the included cLBP patients were otherwise healthy and suffered from low back pain with a minimum of 30 mm on a visual analog scale (0–100 mm) during the 12 weeks prior to screening. The exclusion criteria were as follows: receipt of health care advice for headaches within the past year and more than five headache episodes (one or more lasting more than 2 days); neck pain equal to or exceeding 30 mm on a visual analog scale (0–100 mm), headache within the last six weeks; peripheral neurological deficit; spinal fracture, infection, or cancer; previous surgery involving the back region; previous experience with trunk muscle strength testing; performance of exercise more than two times per week or at a competitive level; inability to follow German verbal instructions; and a BMI exceeding 35 kg/m^2^. Participants were asked not to take analgesic drugs, muscle relaxants, or psycho-pharmaceuticals within 2 days of testing. A total of 243 patients were included in the study.

### Experimental protocol

Schedule of assessments and tasks:

Each individual participated in three assessments performed on three different days; testing was performed at approximately the same time each day to control for the effect of circadian rhythms on muscle strength and endurance measures. The second session was conducted 1 to 2 days after the first and the third approximately 6 weeks later. The 6-week interval between the second and third sessions was chosen to examine whether the SEMG measures investigated would remain stable within an interval that is considered the minimum duration of a therapeutic exercise intervention to demonstrate muscle structural changes in reaction to muscle training (Folland and Williams 2007).

The basic procedure followed by all participants was as follows: (1) completion of validated patient-reported questionnaires that were filled out on tablets under supervision of the examiners, and that assessed (a) pain intensity ratings on a visual analog scale, (b) back-related functional health (Roland Morris Questionnaire (RMQ (Wiesinger et al. 1999)), (c) pain-related disablement using the pain disability index (PDI (Chibnall and Tait 1994)), (d) demographic variables, and (e) physical activity (international physical activity questionnaire, IPAQ (Craig et al. 2003)). Following completion of the questionnaires, participants performed (2) a muscle warm-up and familiarization session on a back extension test device followed by at least two maximum isometric back extensions (100% maximum voluntary contraction (MVC)) tests; (3) a 15-min pause for recovery; (4) attachment of electromyographic and accelerometric sensors; and (5) SEMG recording during an 80% MVC sustained trunk extension held for 30 s.

### Instrumentation

Training and supervision of the examiners and the familiarization of test participants with the appropriate performance of various test protocols were emphasized. The instrumentation, procedures, and data processing completed for this study were published in detail previously (Kienbacher et al. 2014a) and are now briefly described:

#### Handgrip strength

This was measured with a handheld dynamometer (Jamar®, Lafayette Instrument Corp, In, USA).

#### Maximum (100% MVC) and 80% back extension dynamometry

Maximum isometric back extensor muscle strength was measured on the F110 extension device (DAVID® Health Solutions Ltd, Helsinki, Finland), and the 80% MVC back extension on the Total Trunk device (Technogym®, Italy) according to the manufacturers’ instructions. This Total Trunk device allows similar test procedures as the F110 extension device but includes a sacral pad instead of a back pad, thus avoiding pressure on the sensors and enabling unrestricted SEMG recording while testing.

#### Surface EMG

**Fig. 1:**
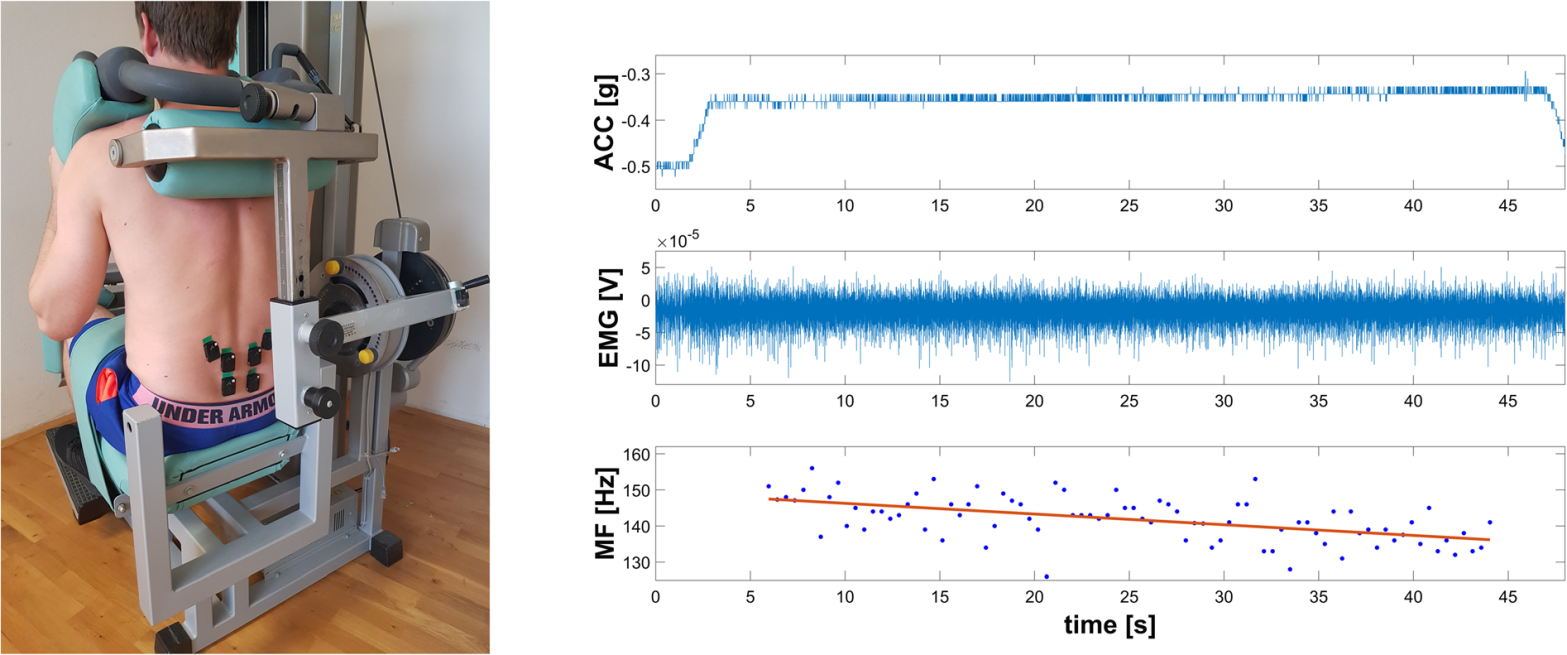
This picture (left) illustrates the positioning of the SEMG sensors (Trigno, DelSys Inc®, Boston, MA, 563 USA) attached when the testees performed the static sustained back extension at 80% of maximum back extension strength using the “Total Trunk” back exercise device (TechnoGym®, Italy). The picture on the right provides an example of the raw signal quality of both the surface electromyogram (upper box), the accelerogram (mid box), and MF-SEMG over time (bottom box) recorded from the L5 left electrode site during an 80% MVC sustained back extension. Data is shown for a representative participant

Electromyographic signals were recorded using active double parallel-bar electrode sensors that also integrated triaxial accelerometric sensors (Model Trigno, DelSys®, Boston, MA, USA). After the skin at the electrode sites was abraded with alcohol and, if necessary, shaved, the electrodes were positioned bilaterally over the multifidus muscle at L5, the longissimus at L2, and the iliocostalis lumborum muscle at L1. The position of the electrodes considered muscle fiber direction and the positioning recommended by the SENIAM project (Hermens et al. 1999) and other previous studies (e.g., Lariviere et al. 2002a, b). Landmark locations rather than the multifidus muscle itself served to establish validity of the SEMG signal, as it is difficult to capture the multifidus muscle with surface electrodes. A reference electrode is not necessary with the Trigno EMG system. The SEMG signals were acquired at a total effective gain of 909 V/V ± 5%, a bandwidth of 20–450 Hz, and a baseline noise < 0.75 μV (RMS). The SEMG signals were sampled at 2000 Hz using a 16-bit AD/board and EMG works acquisition software (DelSys®, Inc., Boston, MA, USA). All sensors were secured to the skin by double-sided adhesive interfaces. To monitor mechanical positioning data during the sustained 80% back extension, one additional sensor was attached to the lever arm of the Technogym device in a standardized way. Figure 1 illustrates the experimental setting.

#### Questionnaires

Pain intensity was rated on a visual analog scale (VAS) ranging from 0 (no pain) to 100 (highest pain) (Sindhu et al. 2011).

The Roland Morris disability questionnaire (RMDQ) comprises of 24 items which patients either agree (1) or disagree (0) with, and the sum score ranges from zero to 24 with higher scores indicating higher disability levels. (Roland and Fairbank 2000)

The Pain Disability Index (PDI) assesses experienced pain-related disability in seven domains of life measured on an 11-point scale (0 = no disability, 10 = total disability). The sum score ranges between zero and 70 with higher scores indicating higher disability.

The International Physical Activity Questionnaire (IPAQ) long-form interview comprising 27 items was used to assess physical activity levels in different life domains (domestic/gardening, work, transport, recreation/sports) (Craig et al. 2003). The weekly average activity levels were converted into metabolic equivalent of tasks (MET) with one MET equating the energy consumption in an inactive state (= 3.5 ml O_2_ kg-1 min-1).

### Test procedures

#### Maximum back extension test

The test leader adjusted the lever arm attachments to the participants’ body dimensions following the manufacturer’s recommendations. The participants were securely fixed with all the restraining DAVID device mechanisms. Thereafter, they performed a warm-up at low loads to familiarize themselves with the equipment and test procedures. This was followed by two consecutive maximum isometric back extensions under supervision of the tester. Intervals between maximum test repetitions were a minimum of 15 s. If the two tests varied by more than 10%, or if the peak moment was achieved later than 3 s after the onset of the contraction, further trials were permitted until a consistent maximum was achieved. The best value obtained was recorded and stored. Verbal instructions and encouragement were standardized.

#### Sustained back extension test

After the electrodes were attached to the muscles of interest and checked for function, study participants were seated on the TOTAL TRUNK device using the same positioning variables that were used for the DAVID® device. After adjusting the lever arm attachments and securing the participant with all restraining mechanisms, the lever arm was loaded with 80% of maximum. With support of the tester, the participant moved her/his trunk into a 20° anteflexed trunk position. In this position, the participant was encouraged to resist the load and to maintain the position for at least 30 s. The 80% maximum voluntary contraction (MVC) load in kilograms was calculated from the best maximum trunk extension moment (Nm) obtained from the DAVID® device. This measure was obtained from the mathematical product of the moment as recorded by the load cell of the dynamometer and the moment arm, as defined by the distance between the back restraint and the load cell.

### Signal processing

#### SEMG signal

MATLAB routines (The MathWorks, Inc., Natick, MA, USA) were used for EMG data processing. All data was preprocessed by skipping the first 3 s of the static task and by removing artifacts. SEMG data was filtered using a 20 Hz high-pass and 500 Hz low-pass Butterworth filter design. Fourier transformation was performed using a Blackman window, epoch 500 ms, 50% overlap resulting in 27 s of data at 2 Hz (i.e., 54 samples for each data set) (Kienbacher et al. 2014a). A linear regression analysis was performed on MF-SEMG data for each electrode site separately between 3 s and 30s of the contraction in order to calculate the rate of decline in MF over time. The slope of the linear regression line was measured in Hz/s and after normalizing to the initial MF value (intercept of the regression analysis), in %/s. Figure 1 illustrates the SEMG signal processing process.

#### Mechanical signal

To control the mechanics of the sustained position task, a triaxial accelerometer (Trigno, DelSys Inc®, Boston, MA, USA) attached to the lever arm of the dynamometer monitored the trunk position during the sustained back extension. The signal was preamplified with a dynamic range of ± 1.5 g and a bandwidth from DC to 50 Hz. Signal sampling occurred at 160 Hz with a resolution of 8 bits using the EMG Works® acquisition software. The orientation of the lever arm of the Total Trunk device was considered as a proxy of the trunk angular displacement and was estimated using the formula acc = asin(acc/g), where “asin” denotes the arcsine (inverse sine function), “acc” is the acceleration measured along the z-axis of the accelerometer during 30s positioned on the Total Trunk device, and “g” is the gravitational acceleration.

#### SEMG ratios and imbalance parameters

Following previous suggestions (Oddsson and De Luca 2003), we calculated an MF ratio for each pair of SEMG electrodes at the three lumbar levels (L1, L2, and L5), for a total of three MF ratios. These variables were calculated separately for each lumbar level from the sample-by-sample ratio (right-side value divided by left-side value) of the two signals of interest between 3 s and 30s of the contraction (for a total of 54 ratios from 27 s of data sampled at 2 Hz). Each of the ratio values was further transformed to provide a time series corrected ratio (R) with symmetrical properties centered around 0. An average of all the transformed ratios was used to represent the segmental imbalance behavior between the two SEMG signals. The derived ratio was expressed as a percentage of the difference between the right and left sides. From these local segmental ratio parameters, two global SEMG parameters, the “uncompensated” and the “compensated” imbalance parameters, were then calculated. The “uncompensated” imbalance was defined as the mean across the three lumbar levels of the absolute value of the segmental ratios, and the “compensated” imbalance as the mean of the segmental ratios across all lumbar levels (Oddsson and De Luca 2003). The uncompensated imbalance parameter thus provides a measure of the total muscular imbalances regardless of direction (right or left), whereas the compensated imbalance parameter takes into consideration the direction of the local segmental imbalances, with a positive value indicating that right > left and a negative value indicating that left > right (Oddsson and De Luca 2003; Oddsson et al. 1997).

### Statistical analysis

#### Definition of variables

The following dependent variables were used in the analysis: MVC as an indicator of back extensor strength; individual mean bilateral IMF-SEMG values as derived from the onsets of the linear regressions; the bilateral MF-SEMG slopes (changes over time) normalized to the onsets of the MF-SEMG based on the three muscle sites to indicate the neuromuscular fatigue rate (L1—iliocostalis lumborum, L2—longissimus, and L5—multifidus); and the uncompensated and compensated values of individual MF-SEMG muscular imbalances. We continuously monitored the trunk position angle and respective changes in trunk position angle during the 30 s static back extension test for quality control of the mechanic performance of the test. The independent variables were participants’ age (young and old) and sex. All statistical analyses were done using R environment for statistical computing® (R Core Team 2019) using the lme4 package (Bates et al. 2015).

#### Sample size estimate

A Monte Carlo power simulation was used to estimate the sample size for the mixed ANOVA (Liu 2013). These simulations were based on the effect sizes observed from a similar study performed using healthy volunteers by our research group; this prior study was of sufficient power to detect medium age-specific effects, but of insufficient power to detect small to medium sex-specific effects (Kienbacher et al. 2014a) as demonstrated in previous research (Clark et al. 2003). In addition, the sample size calculation also considered differences in psycho-emotional features of cLBP participants that might affect their willingness and motivation to provide their best possible back extension performance (Lariviere et al. 2010). Fear avoiders would likely be more reluctant to provide a true maximum than endurers, and such divergence is known to effect the decay of the median frequency fatigue slopes if recorded at a given submaximum load (Oddsson and De Luca 2003). Considering a total of five comparisons at an alpha of 0.01 and a power of 0.9 (1-beta) and a loss of 10% of SEMG signals due to major recording artifacts, a sample size of 240 participants with cLBP was necessary to detect small to medium age- and sex-dependent effects between groups. A supplementary Figure (Figure 1 supplementary) provides a graphic illustration of the simulation considering different effect sizes.

#### Age and sex-specific group differences in EMG fatigue

Linear mixed-effect models including data from all three test days were carried out on each outcome variable with “age,” “sex,” and “test number” as fixed factors and the random factor “persons,” and evaluated whether or not the main outcome variables “MF-SEMG onsets” and “MF-SEMG-fatigue normalized to onsets” differed between the two age- and sex-specific groups. *p* values were adjusted to multiple comparisons (3 electrode pairs with a total of 5 comparisons) and a Bonferroni corrected *p* ≤ 0.01 was considered significant. In addition to the *p* values, Cohen’s *d* values are provided for the main comparisons (Cohen 1988) with *d* > 0.2 indicating small effects, *d* > 0.5 indicating medium effects, and *d* > 0.8 indicating large effects, respectively. Post hoc analyses using estimated marginal means (EMMs) (Lenth 2018) were performed if the effects “age” or “sex” or their respective interaction reached a level of significance. The reported *p* values for these post hoc analyses were adjusted by Bonferroni-Holm correction for different subgroup comparisons and *p* < 0.01 was considered significant.

Quality performance variables were statistically investigated in an explorative way. Thus, *p* values required an adjustment for the two main comparisons that tested for age- and sex-specific effects; a *p* < 0.025 was deemed significant. Unpaired *t* tests investigated whether the MF-SEMG slopes differed from zero. Bonferroni correction considered the five electrode sites and the four age- and sex-specific subgroups and *p* < 0.0025 was considered to be significant.

#### Reliability of SEMG fatigue measurements

Generalizability Theory (G-Theory) (Brennan et al. 2001) was used to examine reliability of the SEMG measures as it considers different sources of measurement error. Using a “multi-factorial random-effects ANOVA” model that included several sources of measurement error related to “subject, day, side, subject X day, and subject X side,” the absolute SEM values as well as the respective coefficients of dependability (D)—which is a type of intraclass correlation coefficient (ICC) with the corresponding absolute error variances and, consequently, the absolute standard error of measurements—were calculated.

## Results

Out of 243 cLBP patients, a total of 229 (112 older than 50 years old; 114 females) completed the experiments on all three tests days. Tests were repeated after 5.4 (± 0.7) and 51.9 (± 1.1) days respectively. The demographic and anthropometric variables are provided in Table 1. Isometric maximum back extension strength was significantly greater in males than females, but similar between younger and older male or female participants. Back pain intensity during the past month or on the day of assessment as well as back pain–related disablement was found to be moderate. There were no age- or sex-specific differences observed for either pain or pain-related disablement. However, back-related functional health as assessed by the RMDQ was significantly more impaired in older as compared to younger participants but did not differ between males and females, respectively.

**Table 1:** Demographic variables and variables related to the biomechanics of test performance. *p* values are provided for differences between age- and sex-specific groups. *p* values were adjusted to multiple comparisons (age- and sex-specific effects), *p* < 0.025 was considered significant. Significant *p* values are given in italics

	18–50 Mean (SE)	50–90 Mean (SE)	Men Mean (SE)	Women Mean (SE)	< 50 vs > 50*	m vs w*
*n*	102	103	102	103	-	-
Women	44	59	0	103	-	-
Age (years)	34.44 (1.34)	64.56 (1.04)	48.31 (2.47)	51.78 (2.30)	*< 0.001*	0.300
BMI (kg/m^2^)	24.21 (0.42)	26.72 (0.46)	26.35 (0.40)	24.35 (0.53)	0.345	*< 0.001*
Pain (last month)	49.03 (1.35)	52.35 (1.89)	50.28 (1.51)	50.66 (1.42)	0.157	0.854
Pain (at test day)	38.02 (1.21)	39.85 (1.49)	38.18 (1.21)	39.79 (1.46)	0.343	0.392
RMDQ	5.48 (0.39)	8.56 (0.50)	7.29 (0.38)	6.40 (0.53)	*< 0.001*	0.172
PDI	13.85 (1.34)	16.87 (1.15)	16.87 (1.35)	13.95 (1.21)	0.088	0.106
IPAQ (MET/ wk)	4431.15 (514.68)	4243.19 (414.39)	3821.75 (393.43)	4892.38 (499.90)	0.773	0.095
Back extension strength (Nm)	208.77 (10.27)	184.71 (9.61)	264.69 (7.90)	143.29 (4.28)	0.086	*< 0.001*
Grip strength (kg)	40.17 (1.70)	30.84 (1.49)	46.98 (1.00)	25.60 (0.71)	*< 0.001*	*< 0.001*
Position (deg)	25.39 (0.82)	21.07 (1.12)	23.51 (0.94)	23.27 (0.95)	*0.002*	0.860
Position change (deg/s)	0.07 (0.01)	0.03 (0.01)	0.07 (0.01)	0.04 (0.01)	*0.015*	0.099

*p value

Considering variables of biomechanical performance of the fatigue test, trunk flexion at the beginning of the task was more inclined in younger as compared to older participants, but did not differ when compared between the two sex groups. With the duration of the 30 s of sustained back extension, individuals of all groups shifted their trunks slightly more into an ante-flexed position. Such trunk inclination was significantly larger in males and in younger cLBP individuals than in their respective counterparts (Table 1).

### MF-SEMG fatigue

The MF-SEMG onsets obtained from the sustained back extensions were highest in L5 and lowest in those electrode sites that revealed the most negative fatigue slope. There were no age-specific differences found, but significant sex-specific differences were observed at all electrode recording sites. Cohen’s *d* values indicated moderate to small effects. The variable “test day” revealed significant effects for L1 and the most negative electrode (Table 2).

**Table 2:** Results of “age-” and “sex-” specific effects calculated for the MF-SEMG using linear mixed effect models that also considered the test day recorded from 225 individuals with chronic LBP who performed a stustained back extension at 80% of maximum. Note that *p* values were adjusted to multiple comparisons (3 electrode pairs, 5 comparisons) using Bonferroni correction; a *p* < 0.01 was considered significant. We only indicate * for significant MF-SEMG fatigue slopes for *p* < 0.0025)

	Mean (SE)				Linear mixed effects model						
Level/*n*	< 50	> 50	m	w	Age *F*; *p*	Age *d*	Sex *F*; *p*	Sex *d*	Age × sex *F*; *p*	Age × sex *d*	Test day *F*; *p*
MF onsets (Hz; *n:* 435)											
all	100.98 (0.97)	101.91 (1.11)	97.27 (0.98)	105.57 (1.09)	0.00; 0.971	0.01	*19.54; < 0.001*	0.62	*8.85; 0.003*	0.42	1.19; 0.305
L5	116.64 (1.27)	120.41 (1.37)	113.44 (1.10)	123.99 (1.41)	1.35; 0.247	0.16	*15.33; < 0.001*	0.55	*7.83; 0.006*	0.39	0.26; 0.772
L2	101.55 (1.24)	99.35 (1.42)	95.57 (1.22)	105.38 (1.30)	1.60; 0.208	0.18	*19.09; < 0.001*	0.62	*6.71; 0.010*	0.37	0.04; 0.963
L1	85.51 (0.80)	86.04 (1.05)	83.13 (0.97)	88.14 (0.93)	0.01; 0.924	0.01	*12.56; < 0.001*	0.50	6.55; 0.011	0.36	*8.70; < 0.001*
m.n.	79.15 (0.79)	80.15 (1.03)	77.08 (0.92)	81.99 (0.86)	0.00; 0.973	0.00	*11.27; < 0.001*	0.47	6.20; 0.014	0.35	6.78; 0.001
un.imb.	8.69 (0.39)	9.23 (0.40)	9.30 (0.40)	8.62 (0.39)	1.62; 0.205	0.19	0.76; 0.385	0.13	2.01; 0.158	0.21	0.52; 0.595
c.imb.	− 4.14 (0.51)	− 2.62 (0.62)	− 2.64 (0.67)	− 4.05 (0.48)	5.67; 0.018	0.35	2.99; 0.085	0.29	0.32; 0.574	0.08	0.00; 0.997
MF changes normalized to Onsets (%/s; *n:* 435)											
all	− 0.21 (0.01)*	− 0.12 (0.01)*	− 0.19 (0.01)*	− 0.13 (0.01)*	*14.78; < 0.001*	0.56	5.24; 0.023	0.33	0.61; 0.436	0.11	0.57; 0.567
L5	− 0.19 (0.01)*	− 0.11 (0.01)*	− 0.18 (0.01)*	− 0.11 (0.01)*	*12.91; < 0.001*	0.53	*9.77; 0.002*	0.46	0.63; 0.427	0.12	0.95; 0.389
L2	− 0.23 (0.02)*	− 0.12 (0.01)*	− 0.21 (0.02)*	− 0.14 (0.01)*	*15.32; < 0.001*	0.57	4.00; 0.047	0.29	0.05; 0.828	0.03	0.13; 0.876
L1	− 0.19 (0.01)*	− 0.12 (0.01)*	− 0.18 (0.01)*	− 0.13 (0.01)*	*7.26; 0.008*	0.39	1.08; 0.299	0.15	1.03; 0.313	0.15	2.10; 0.125
m.n.	− 0.38 (0.02)*	− 0.26 (0.02)*	− 0.37 (0.02)*	− 0.27 (0.02)*	*12.13; 0.001*	0.51	5.75; 0.017	0.35	1.41; 0.236	0.17	0.87; 0.419

*m.n.*, most negative electrode; *un.imb.*, uncompensated imbalances; *c.imb.*, compensated imbalances; *p*, *p* value (significant *p* values are given in italics); *F*, *F-value*; *d*, Cohen’s d

*p < 0.0025 (Bonferroni corrected significance level)

**Fig. 2:**
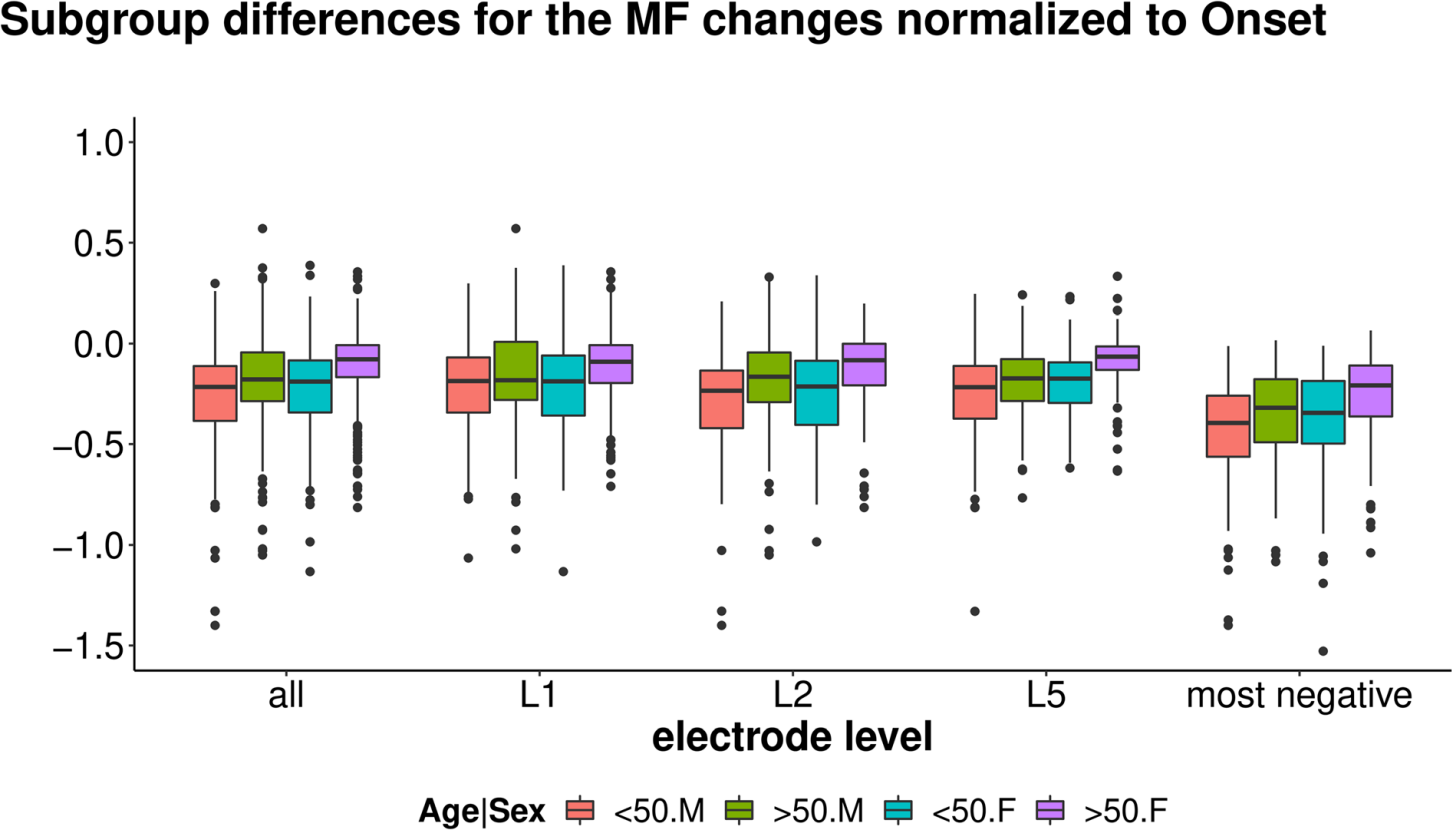
Graphic representations of the post hoc analyses of the MF-SEMG fatigue normalized to onsets. This figure shows the simple box plots of the MF-SEMG fatigue slopes normalized to their onsets (%/s) that were recorded from cLBP patients during a 30 s sustained back extension test performed in a seated position. The box plots are provided for the different electrode recording sites according to the four different groups investigated. These groups were the male cLBP participants younger and older than 50 years of age (“< 50.M”,“> 50.M”), and the female cLBP participants younger and older than 50 years of age (“< 50.F” or “> 50.F”). Please note that results of the unpaired *t* tests suggest that the MF-SEMG slopes significantly differed from zero at all electrode sites investigated and for all the age- and sex-specific subgroups (*p* < 0.0025). Please see also Table 2

The MF-SEMG slopes normalized to the MF-SEMG onset values significantly decreased with the duration of the sustained back extension, and these slopes were significantly more pronounced in younger than in older cLBP individuals with Cohen’s *d* values indicating medium effects at all recording sites except for L1. Regarding sex-specific differences, male participants demonstrated significantly more MF-SEMG fatigue in L5 and a tendency toward significance if the electrode with the most negative slope or if all electrodes were considered. The variable “test day” did not exhibit any significant effects. Post hoc analyses revealed that the age-specific differences were particularly pronounced in females at all electrode sites, and that the sex-specific differences were confined to older individuals and the L5 electrodes only (Table 3 and Fig. 2). Considering imbalances, the compensated MF-SEMG fatigue imbalance scores were significantly more lateralized to the left side (more negative scores) in younger as compared to older participants. Likewise, a tendency toward more lateralization to the left was also observed in male than in female individuals with cLBP. The results of the age- and sex-specific differences of the MF-SEMG are presented in Table 2.

**Table 3:** Results of the post hoc analyses of the MF-SEMG fatigue changes normalized to onsets using estimated marginal means (EMMs) observed from the different electrode pair recording sites, from all electrodes, and the electrode demonstrating the most negative MF-SEMG slope. Note that *p* values were adjusted to multiple comparisons (3 electrode pairs, 5 comparisons) using Bonferroni correction; *p* < 0.01 was considered significant. Significant values are given in italics

	Sex	Difference mean (SE)	< 50 vs > 50 *p* value	Age	Difference mean (SE)	Males vs females *p* value
All electrodes	M F	− 0.07 (0.03) − 0.11 (0.03)	0.036 *0.001*	< 50 > 50	− 0.04 (0.03) − 0.07 (0.03)	0.287 0.031
L5	M F	− 0.06 (0.03) − 0.10 (0.03)	0.050 *0.002*	< 50 > 50	− 0.05 (0.03) − 0.09 (0.03)	0.101 *0.006*
L2	M F	− 0.11 (0.04) − 0.12 (0.04)	*0.009* *0.004*	< 50 > 50	− 0.05 (0.04) − 0.07 (0.04)	0.209 0.119
L1	M F	− 0.05 (0.04) − 0.10 (0.04)	0.238 *0.009*	< 50 > 50	− 0.00 (0.04) − 0.06 (0.04)	0.984 0.149
Most negative	M F	− 0.07 (0.05) − 0.15 (0.05)	0.108 *0.001*	< 50 > 50	− 0.03 (0.05) − 0.11 (0.05)	0.393 0.012

### Reliability

The absolute MF-SEMG values were varied and found to be greatest in L1, and smallest if all electrodes were considered. Of note, the relative SEM values expressed as a percentage of the MF-SEMG onsets were clearly lower than 10% in all of the different recording sites (data not shown). Considering the normalized MF-SEMG slopes, the SEM values were varied. The respective SEM values of this variable relative to the slopes exceeded 10% in all of the electrode sites investigated and there were no major differences between the two age-specific subgroups.

Relative reliability as derived from *d* values obtained for the MF-SEMG onsets at all the different recording sites exceeded values of 0.75, indicating excellent relative reliability with no major differences between younger and older cLBP patients. The respective values for the MF-SEMG slopes were overall excellent with values in a range between 0.78 (L1, < 50 years old) and 0.92 (L5; > 50 years old), respectively. There were no relevant differences between younger and older individuals observed for these ICC values. All the ICC and SEM values calculated for the different SEMG recording sites are provided in Table 4.

**Table 4:** Test-retest reliability of the main outcome variables derived from the MF-SEMG recorded on three different test days

	MF			
	Onset		Change/s (normalized)	
	*D*^1^	SEM	*D*^1^	SEM
All electrodes	0.786	6.652	0.923	0.044
< 50	0.795	6.224	0.906	0.047
> 50	0.780	7.054	0.924	0.041
L5	0.909	4.774	0.835	0.065
< 50	0.898	4.788	0.795	0.073
> 50	0.913	4.821	0.849	0.056
L2	0.899	5.007	0.827	0.079
< 50	0.880	5.391	0.810	0.082
> 50	0.904	5.012	0.839	0.069
L1	0.859	4.488	0.797	0.077
< 50	0.838	4.555	0.777	0.080
> 50	0.877	4.405	0.778	0.074
Most negative	0.860	4.518	0.877	0.064
< 50	0.854	4.395	0.853	0.068
> 50	0.866	4.623	0.874	0.060

^1^*D* = *D* value

## Discussion

This study is among the first to investigate how advancing age alters the neuromuscular capability and associated muscle glycolytic metabolism of the back extensor muscles in male and female individuals with cLBP. Using the MF-SEMG back muscle fatigue method, major findings of this research revealed, despite some significant differences in the biomechanical performance markers of the tests, that:

1. MF-SEMG fatigue was significantly less in older as compared to younger cLBP individuals, and such differences were more pronounced in females than in males;
2. MF-SEMG fatigue was significantly larger in older male as compared to female cLBP participants; and,
3. Relative reliability was excellent for the MF-SEMG fatigue slopes, but absolute reliability indicated large test-retest variability for both of these SEMG variables.

### Methodological considerations

Before comparing the MF-SEMG fatigue findings between the age- and sex-specific subgroups, it is important to verify group equivalence for the mechanical recording variables of the fatigue test derived from a loaded trunk position test as administered in this study. Prior studies suggest that MF-SEMG fatigue changes are highly dependent on the load relative to an individual’s true maximum (e.g., Lariviere et al. 2010; Oddsson and De Luca 2003). As cLBP individuals might be reluctant to perform a true maximum back extension due to fear of injury or fear of pain exacerbation (Vlaeyen et al. 2016) or other psychological factors (Lariviere et al. 2010), performance of a close to true maximum back extension torque in all subgroups is of utmost importance to obtain reliable results if the SEMG monitors of fatigue recorded from submaximal sustained contractions are to be compared. Because no objective measures seem to exist for a true maximum back extension torque production, we compared the scores obtained from our cLBP sample with those from two historic samples of healthy younger and older male and female study participants that had been tested using similar equipment (Kienbacher et al. 2014b; Smith et al. 1985). The maximum strength scores obtained from both our younger and older male and female cLBP participants were (by direct comparison of the means) approximately 5 to 10% smaller than those observed from previously tested healthy controls. Considering this, the 80% target load used for the sustained contraction was likely based on close to true back extension maximum scores in all the cLBP subgroups tested in this study, a requirement that allows for confident comparisons of the SEMG findings observed between groups. This conclusion is further supported by the significantly smaller handgrip strength values relative to the non-significantly smaller back extension values observed in the older participants of this study, as well as lack of a significant root mean square (RMS)-SEMG increase over the duration of the test, when all electrodes were considered (data not shown).

At the beginning of the fatigue tests, participants had their trunks ante-flexed at an average lever arm angle of approximately 21 degrees. As previous research has found the maximum back extensor torque production to increase with an increase of trunk inclination (Graves et al. 1992), the larger trunk inclination angle of 4 degrees in the younger participants of this study suggest that the torque production of their back extensors may have been relatively smaller during the sustained contraction than that observed in their older counterparts. This is because the load used for the fatigue test equated 80% of maximum that was obtained from a maximum force task recorded with all participants in a 30° anteflexed trunk position. Consequently, one might expect slightly smaller MF-SEMG changes in younger as compared to older individuals, indicating less muscle fatigue due to differences in trunk position and relative load. However, as MF-SEMG fatigue was more pronounced in younger than in older participants, such differences would be unlikely to confound the interpretation of our SEMG findings.

Although participants were able to keep the position of the 80% MVC load relatively constant during the sustained extension, slight increases in trunk flexion were observed. These were significantly more pronounced in younger than older and in male than female participants, respectively. Inability to maintain a given position likely indicates fatigue-related contraction failure, which suggests that back muscle fatigue occurred earlier and to a stronger extent in the younger and particularly male cLBP participants. Whereas such between-group differences might be assumed to confound a SEMG signal with a small signal to noise ratio (high noise), as this was the case in low force back extensions (Lariviere et al. 2002a; Lariviere et al. 2009), the signal to noise ratio was high in our back extensions that were close to a maximum. Consequently, it can be concluded that the differences in trunk inclination changes with the duration of the sustained contractions were unlikely confounded by noise, and thus the group comparisons of the SEMG fatigue changes can be relied upon.

### MF-SEMG changes with fatigue

The main focus of this research was on the MF-SEMG fatigue changes performed at the 80% sustained back extension. Assuming that local muscle blood flow is occluded at contraction strengths exceeding 50 to 60% MVC, and that spatial filtering due to inter-individual differences in electrode muscle configuration would be unlikely to affect the changes over time of the SEMG frequency content (for a discussion see (Ebenbichler et al. 2017), this measure captures the physiologic changes in a muscle that occur with fatigue. A slowing of the muscle fiber conduction velocity within the fast fatiguing, glycolytic muscle fibers which compose the high threshold MUs is believed to relate to the metabolic byproduct accumulation of lactate and K+ with muscle fatigue (Brody et al. 1991; Kupa et al. 1995; Roy et al. 1997). Consequently, if other confounders that might affect the MF-SEMG changes are well controlled for, as it is the case in this study, an increase in muscle fatigue likely relates to an increase in the proportion of active fast fatiguing glycolytic muscle fibers (De Luca 1997; Merletti et al. 2002; Merletti et al. 1992; Roy et al. 1997). Consistent with these assumptions, findings of this research observe a significant decrease of the MF-SEMG with the duration of the sustained back extension in all the different electrode sites. This decrease was significantly smaller in older than in younger cLBP individuals overall, thus likely indicating that a smaller relative percentage of active glycolytic muscle fibers was available to contribute to this task. Although such significant slopes in MF-SEMG back fatigue have repeatedly been observed in previous research in cLBP patients (e.g., da Silva et al. 2015; Davarian et al. 2012; Roy and Oddsson 1998), we, for the first time, were able to demonstrate that there was a difference if the individuals’ age was considered. This suggests that the aging process in back extensor muscles remains widely unaffected even in the presence of potentially antagonizing muscle metabolic processes that may occur with cLBP. Our findings of a decreased glycolytic muscle metabolic state in older as compared to younger cLBP individuals provides muscle function information that complements findings of structural changes obtained from muscle probes with a smaller glycolytic type II fiber proportion in older as compared to younger individuals with and without cLBP (Mannion 1999; Mannion et al. 1998; Ng et al. 1998). Our findings also complement radiologic imaging studies that demonstrated an increased muscle fat content that preferably affected the multifidi muscles in older compared to young cLBP individuals (Sions et al. 2017a), and that was more pronounced in female than male patients with lumbar spine pathology (Shahidi et al. 2017). It is important to note that such additional information on muscle function as derived from spectral SEMG fatigue to MRI back muscle imaging—both examinations are offered to cLBP patients in clinical practice—would not allow one to distinguish between different underlying causes of the altered MF-SEMG fatigue in older cLBP patients. Such causes would have to be ruled out by other diagnostic techniques that identify the occurrence and shape of individual motor units (diagnostic EMG). Both an increased intramuscular fat content accompanied by a decreased SEMG fatigue could result from either non-recruitable, denervated muscle fibers in consequence to axonal degeneration with a consecutively increased proportion of type I fibers because of neuromuscular regeneration, or from long-term recruitment failure of high threshold type II glycolytic muscle fibers (McGregor et al. 2014). The former likely suggests degeneration followed by secondary (incomplete) regeneration (McGregor et al. 2014), and the latter, long-term impairment in neural drive with secondary inactivity-driven adaptations in muscle fibers (Chiou et al. 2014). Previous research into normative values for paraspinal denervation using needle EMG showed older asymptomatic study participants had more denervation than younger ones (Tong et al. 2005).

Our observations of significant age effects in individuals with cLBP seem to contrast those of recent research examining MF-SEMG fatigue of the back extensors (da Silva et al. 2015). The findings of this previous study did not observe any age-specific effects, neither in healthy volunteers nor in cLBP individuals, although significantly steeper MF-SEMG slopes were observed in cLBP participants as compared to healthy controls. The fatigue test conducted in this previous study was performed at 50% of the voluntary back extension maximum, a load that might be insufficient to recruit all MUs and occlude back muscle blood flow in all individuals (De Luca 1997). In addition, a Roman chair was used for the fatigue test with the participants in a semiflexed prone lying position and shanks fixed. As compared to our study procedure, a Roman chair back muscle fatigue test performed at 50% MVC would likely be more prone to confounders of the SEMG signal. At 50%, the blood flow in the back muscles may not be fully occluded thereby affecting MF-SEMG fatigue signal in a variable way. Testing of individuals in a sitting position as is done using the TECHNOGYM or DAVID device likely challenges the back extensor muscles more specifically than the Roman chair test could, thereby introducing less variability (Lariviere et al. 2011). Indeed, the trunk extension force production in a Roman chair is achieved through the shared contributions of the biceps femoris, hip, and back extensors that are tightly coupled via the fascia thoracolumbalis and ligamentum sacrotuberale (Vleeming et al. 1995; Willard et al. 2012). Differences in synergistic muscle recruitment patterns between the different age groups would be able to cover any age-specific effects in back SEMG fatigue.

Our study’s results revealed that less MF-SEMG fatigue occurs in the multifidus muscle, and that muscles demonstrate the most MF-SEMG fatigue in older females, as compared to older males. Such sex-specific differences suggest substantial differences in the glycolytic metabolism of the back extensor muscles. These observations appear consistent with findings from histological probes that observed a smaller proportion of glycolytic type II muscle fiber in females as compared to male cLBP patients, and from muscle endurance tests that revealed stronger muscle fatigability in males than in females (Hunter et al. 2016). Sex-specific differences in back muscle metabolism could further reflect differences in pain-related adaptations in motor control strategies of the trunk between males and females, as has previously been observed in healthy pain free individuals (Clark et al. 2003). Such sex-specific differences in motor control could also relate to a higher age-related decrease in trunk flexibility in males than in females (Kienbacher et al. 2016), as well as to an observed increased segmental instability in older females as compared to males. In the presence of back pain, alterations in neuromuscular control of the trunk muscles might be facilitated differently between males and females, which could further drive divergent secondary neuromuscular adaptations consistent with the sex-specific differences in the suggested back muscle glycolytic metabolism observed in this study. In addition, differences in (long-term) neuromuscular adaptations might further be facilitated by sex-specific differences in movement behavior which are strongly driven by reinforcement learning and could reasonably differ between males and females (van Dieen et al. 2017; van Dieen et al. 2018).

### Reliability

Consistent with findings from studies that sought to investigate the reliability of the MF-SEMG fatigue method, the good to excellent values observed in our study suggest that these variables are well suited for distinguishing between muscles with a low and a high muscle glycolytic metabolism (Kienbacher et al. 2014a; Villafane et al. 2016). This method could thus be suggested as a suitable diagnostic tool to diagnose muscle functional features in older individuals with cLBP, or to identify muscle metabolic differences between older male and female cLBP patients. Observations of both comparable maximum back extensions strength scores and physical activity levels as assessed with the IPAQ in our older and younger cLBP participants confirm previous observations that suggested the MF-SEMG fatigue method to be independent from participants’ absolute force generating capacity, as long as a true maximum score served to determine the load of the 80% MVC sustained contraction (Oddsson and De Luca 2003). This further suggests that the MF-SEMG method is a reliable diagnostic/predictive marker that is widely independent from the neuromuscular regenerative and compensatory processes that may occur in aging muscles subsequent to axonal loss (Frontera et al. 2008; Frontera and Ochala 2015).

Also consistent with previous research is the more disappointing absolute reliability as expressed by SEMs which points to a poor capacity for the method to identify changes over time (Kienbacher et al. 2014a; Villafane et al. 2016). Feedback mechanisms that allow for an improved standardization of the mechanic performance may increase absolute reliability of the back extension test. Although participants were positioned in a standardized way, the electrode recording site demonstrating the most negative fatigue slope varied from test day to test day (please see supplementary Figure 2), suggesting that even in a simple, and obviously well-standardized, static back extension test variable neuromuscular activation patterns of the back extensors seem to exist. To further develop the MF-SEMG fatigue method so that it may be used as a surrogate marker in clinical studies, or to monitor treatment outcome in an aging or sarcopenic population, either maximum muscle strength and/or power scores would need to be added to the MF-SEMG fatigue scores. The resulting combined scores, or the baseline MVC scores used to determine the 80% load for the fatigue test, would need to be administered again after completion of a therapeutic intervention.

### Limitations

As sex-specific differences were confined to older females, and age-specific differences were particularly pronounced within the female group, we were careful in considering inadequate “maximum back extensor strength scores” as a potential confounder of these findings. However, the maximum strength scores were comparable not only between the younger and older female but also between the younger and older male cLBP participants, and the observed score of these measures were unaffected by psycho-emotional variables like the “avoidance endurance behavior” subgroups as defined by Hasenbring (Hasenbring et al. 2009) (data not shown). We therefore feel confident that despite the cross-sectional nature of this study with repeated measurements, our results are not biased by this possible confounder.

Participants revealed lower back pain intensity ratings on the day of assessment than they had reported during the prior month, with all reported intensities indicating moderate pain**.** Indeed, pain intensity of cLBP is known to vary and the attention to the test situation may have interfered with pain perception. In fact, the moderate pain scores observed within the month prior to testing are typical for cLBP individuals.

### Implications

Excessive weakening of the back extensor muscles is a known risk factor of LBP. Therefore, long-term back-related disability is a driver of early frailty and the need for institutionalization, particularly in old age (Beauchamp et al. 2016; Schmidt et al. 2017; Suri et al. 2009; Suri et al. 2011). Muscle function–based biomarkers, like the MF-SEMG method, likely allow one to accurately diagnose the very early forms of an accelerated neuromuscular aging process in individuals who are at the age of 50 to 60 years, which might indicate an increase in the risk of sarcopenia in later years. This would hold true not only in healthy, pain-free individuals (Kienbacher et al. 2014a) but also in those with cLBP as suggested by the finding of this study. A smaller decline in MF-SEMG fatigue slopes would relate to less glycolytic muscle activity, thereby indicating a decrease in number and size of type II muscle fibers. Such changes may likely result from both an axonal loss of preferentially highly myelinated axons and a blunted regenerative capability through type II satellite cells and/or incomplete regeneration through incomplete collateral sprouting of orphanized typ II muscle fibers shifting toward type I fibers (for review see (Larsson et al. 2019)). Findings of a recent study performed on both young and old sarcopenic and non-sarcopenic men suggest that both the loss of motor neurons per se, and an impaired collateral sprouting resulting in a failure to expand motor unit size, are critical factors that determine the progression of sarcopenia in old age (Piasecki et al. 2018). In addition, type II muscle fibers are thought to belong to the large motor units with highly myelinated axons that are recruited last in static and concentric contractions at increasing force (Contessa et al. 2016), and have the capacity to generate the highest twitch contraction forces. Thus, the age-related flattening of the fatigue slopes MF-SEMG in older individuals likely indicates an early decrease in both muscle power and muscle strength as a diagnostic and predictive marker rather than muscle endurance function in aging individuals. The rate of MF fatigue would not be affected in a major way even if an awaited decline in mechanic output from muscle strength and power measurements (due to axonal and glycolytic muscle fiber loss) was well compensated (McKinnon et al. 2015). It is worth noting that early declines in muscle power function seem more greatly associated with limitations in activities and mobility than muscle strength (Reid and Fielding 2012; Suetta et al. 2019). Thus, the very early detection of individuals with or without cLBP who are in their 50s or 60s and who would be classified to be at an increased risk for sarcopenia (in their later age) could allow initiation of early interventions in these individuals; this would reduce the health burden of sarcopenia (Calvani et al. 2017; Ciolac and Rodrigues-da-Silva 2016). In addition to its high potential as a diagnostic marker, the MF-SEMG fatigue method could also be administered to monitor treatment outcome, particularly if drug interventions were available that could stop or slow down an early/accelerated neuromuscular aging process. The MF-SEMG method is not a surrogate marker to monitor treatment outcome per se. However, it could further be developed toward becoming a reliable surrogate marker to monitor improvements in deteriorated muscle output through interventions like muscle power training and neuromuscular electrostimulation (Kern et al. 2014), if longitudinal changes in maximum muscle strength/ power scores are considered together with the MF-SEMG fatigue scores.

To determine appropriate intervention settings, subsequent diagnostic tests would have to rule out underlying causes that may explain the variability observed with a SEMG screening test. This would include the examination of (1) an increased axonal loss and regeneration with a concomitant muscle cross-sectional atrophy; (2) secondary muscle metabolic adaptations due to increasing load accompanying a forward-leaning posture in elderly persons; and/or (3) a decrease in neural drive with some high threshold motor units becoming unrecruitable, whereby muscle fibers increase their fat content. Whereas in cLBP it is generally of importance to train all muscle qualities (strength, power, endurance, flexibility) including sensorimotor control, findings from our study suggest that particularly in female cLBP patients, focus should be put on the augmentation of back extensor strength and power through muscle training or neuromuscular electrostimulation relative to other training interventions in order to minimize some aspects of the muscle aging process (Zampieri et al. 2015).

In conclusion, the age- and sex-specific differences in back muscle function observed in a cLBP population with the MF-SEMG fatigue method suggest that the diagnostic potential of this method remains widely unaffected by the neuromuscular changes that may occur with the perception of chronic pain in the lower back. Thus, this method possesses great potential as a predictive and diagnostic biomarker that can detect precursors of an early or accelerated aging process within the neuromuscular system, identifying such individuals at an increased risk for sarcopenia in their later lives. Very early detection of aging back muscles would be relevant to the design of therapeutic exercise programs intended to prevent an early aging process and disablement in older individuals.

## Electronic supplementary material

Figure 1 supplementary Graphic illustration of the Monte Carlo simulation considering different effect sizes. (PNG 42 kb)

High Resolution Image (TIF 3276 kb)

Figure 2 supplementary This figure shows the electrode recording site that revealed the most pronounced/ negative MF-SEMG fatigue slope and the respective changes when the individual was retested on a second or third examination day. The arrows indicate how the electrode site depicting the most MF-SEMG fatigue changed between days. (PNG 1319 kb)

High Resolution Image (TIFF 4106 kb)
