# Supplementary figures and images for: Age- and sex-specific effects in paravertebral surface electromyographic back extensor muscle fatigue in chronic low back pain
Source: GeroScience. 2019 Nov 27;42(1):251–69. doi: 10.1007/s11357-019-00134-7 (PMC7031171; doi:10.1007/s11357-019-00134-7)

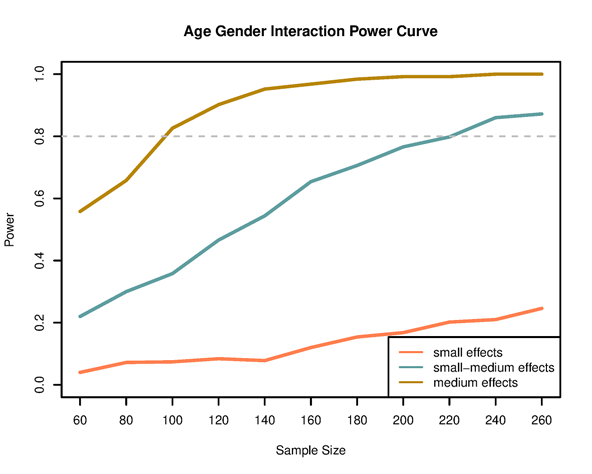

Supplement: Supplementary file 1 — Graphic illustration of the Monte Carlo simulation considering different effect sizes. (PNG 42 kb) [file 11357_2019_134_Fig3_ESM.png]

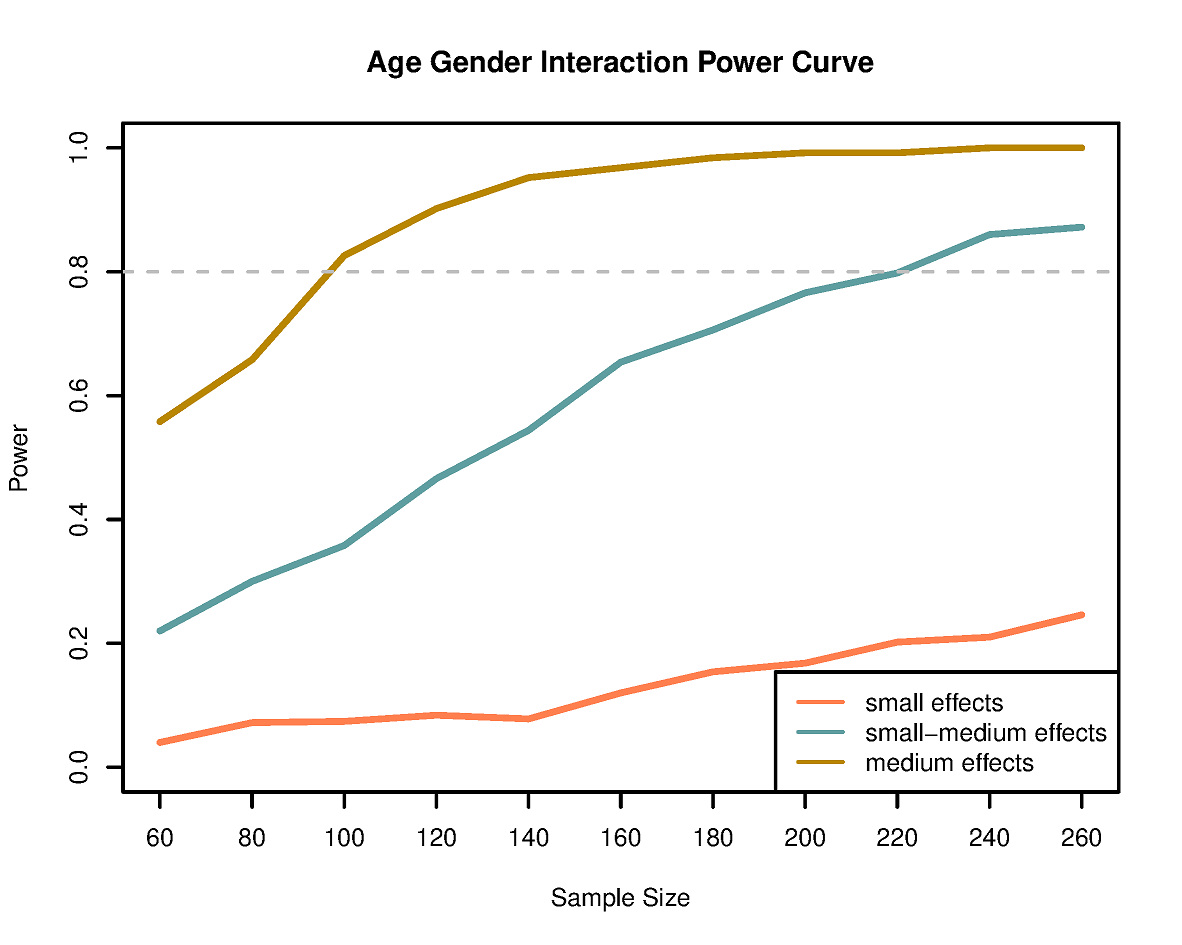

Supplement: Supplementary file 2 — High Resolution Image (TIF 3276 kb) [file 11357_2019_134_MOESM1_ESM.tif]

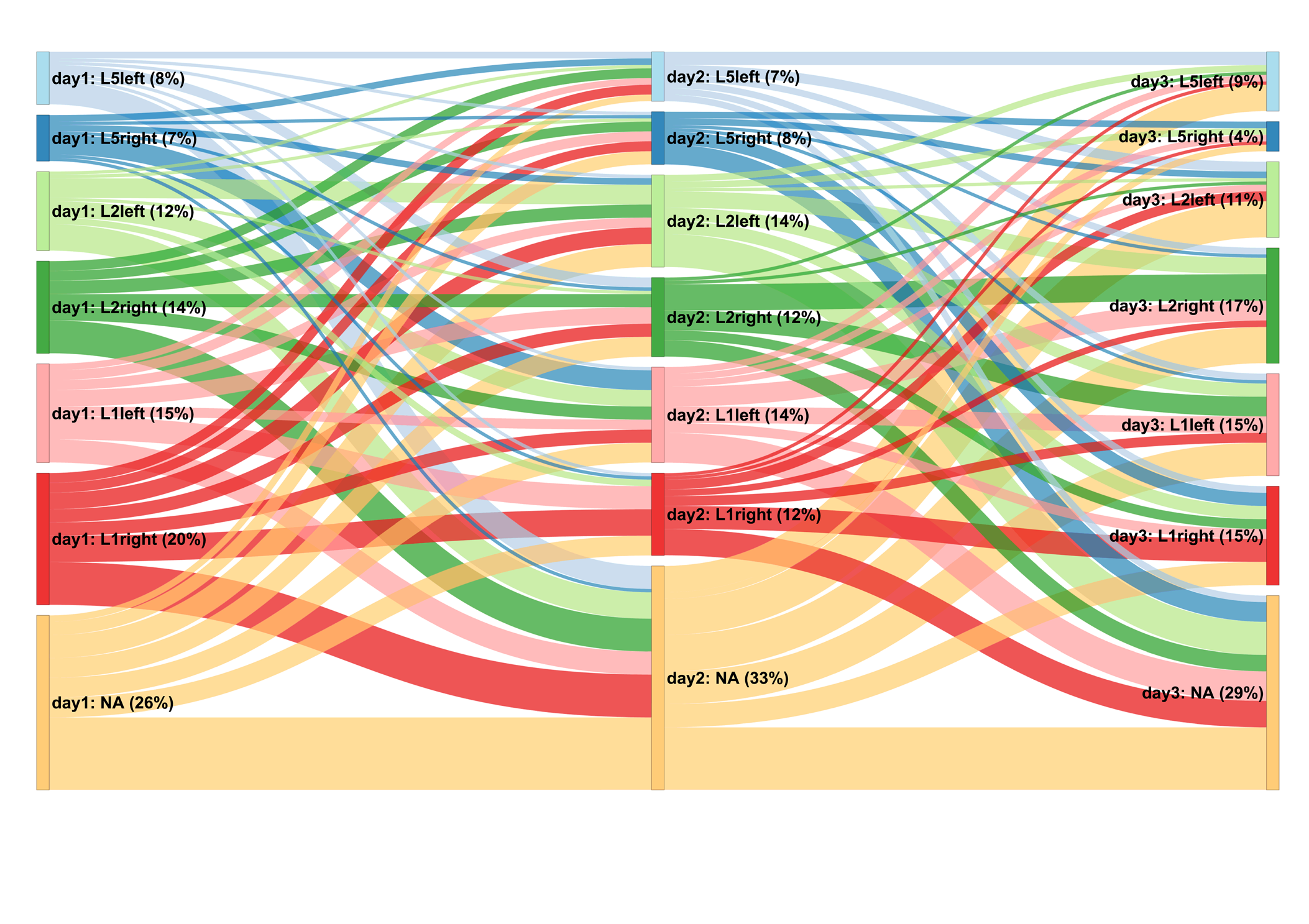

Supplement: Supplementary file 3 — This figure shows the electrode recording site that revealed the most pronounced/ negative MF-SEMG fatigue slope and the respective changes when the individual was retested on a second or third examination day. The arrows indicate how the electrode site depicting the most MF-SEMG fatigue changed between days. (PNG 1319 kb) [file 11357_2019_134_Fig4_ESM.png]

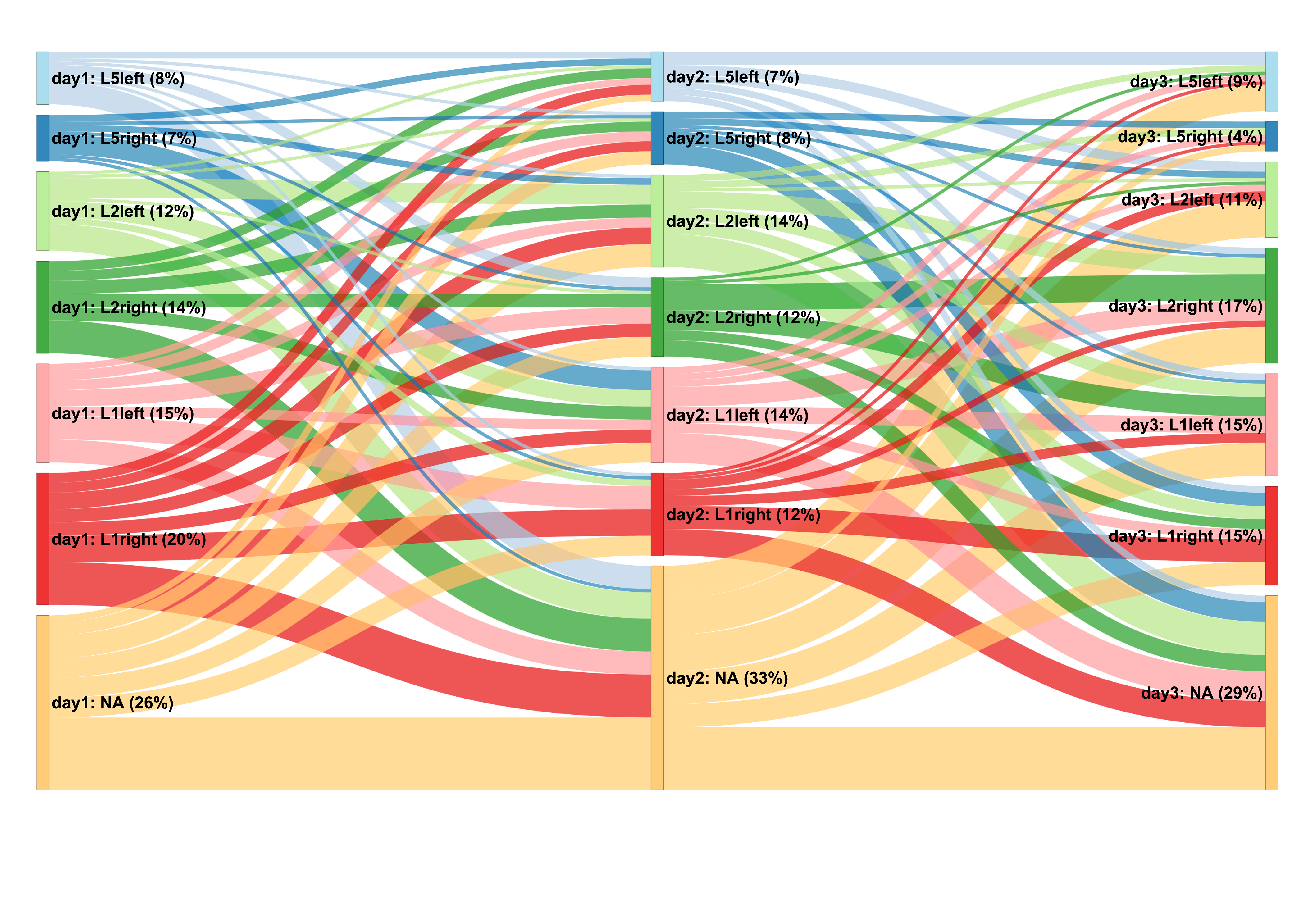

Supplement: Supplementary file 4 — High Resolution Image (TIFF 4106 kb) [file 11357_2019_134_MOESM2_ESM.tiff]
